# A Rash Decision: Mycoplasma-Induced Mucositis in a Young Adult

**DOI:** 10.7759/cureus.103722

**Published:** 2026-02-16

**Authors:** Carlos Fagundo, Taariq Imami, Vladimir Valencia, Sapna Patel, Parul Aneja

**Affiliations:** 1 Internal Medicine, BayCare Health System, Tampa, USA; 2 Infectious Diseases, BayCare Health System, Tampa, USA

**Keywords:** atypical rash, conjunctivitis, mycoplasma pneumoniae, mycoplasma pneumoniae-induced rash and mucositis, oral mucositis

## Abstract

*Mycoplasma pneumoniae*-induced rash and mucositis (MIRM) is a distinct emerging clinical entity typically affecting adolescents and adults after a respiratory infection from *Mycoplasma pneumoniae*. Other known mucocutaneous complications include Stevens-Johnson syndrome, erythema multiforme, and toxic epidermal necrolysis. We present the case of a 24-year-old male patient who developed fever and cough followed by a rapidly evolving mucocutaneous syndrome characterized by oral mucositis, bilateral conjunctivitis, and vesiculobullous skin lesions affecting the palms, soles, and trunk with no evidence of skin detachment. These findings meet the proposed diagnostic criteria for MIRM. This case highlights the importance of recognizing MIRM as a distinct diagnostic entity to guide targeted antimicrobial therapy and avoid unnecessary morbidity associated with misdiagnosis and inappropriate treatment. Early differentiation from Steven-Johnson syndrome and toxic epidermal necrolysis is critical for optimal patient outcomes.

## Introduction

*Mycoplasma pneumoniae* is a common cause of community-acquired pneumonia, especially in pediatric and young adult populations. It is transmitted via respiratory droplets [1]. *M. pneumoniae *has been associated with various extrapulmonary complications, including dermatologic, neurologic, cardiac, and hematologic syndromes [1,2]. Among these extrapulmonary complications, *Mycoplasma pneumoniae*-induced rash and mucositis (MIRM) represents a clinically important manifestation. Mucocutaneous manifestations have been reported in up to 25% of *M. pneumoniae *infections, most commonly in children and young adults [2].

MIRM has been defined as a distinct entity since 2015 [3]. It is different from Stevens-Johnson syndrome (SJS) and toxic epidermal necrolysis (TEN) in its presentation. MIRM is characterized by more mucosal involvement, including ocular and genital areas, with less cutaneous involvement. Compared to SJS and TEN, MIRM has decreased severity of its clinical course [3-5]. Diagnosis is clinical with supportive laboratory findings of positive *M. pneumoniae *or serology polymerase chain reaction (PCR) if appropriate. Treatment is supportive, with antibiotics treating the underlying *M. pneumoniae*. Adjunct corticosteroids or intravenous immunoglobulin (IVIG) can be used for refractory cases [3-5]. Our case adds to the expanding body of literature on MIRM.

## Case presentation

We present the case of a 24-year-old male patient with a past medical history of asthma who presented to the emergency department with a one-week history of fever and cough, followed by the onset of worsening rash over three days. The rash began in the oral cavity and then spread to the palms, soles, and trunk. He reported associated redness of his eyes with crusting around them. He also reported dysphagia. The patient denied any recent antibiotic use, high-risk sexual behavior, or sick contacts.

**Figure 1:**
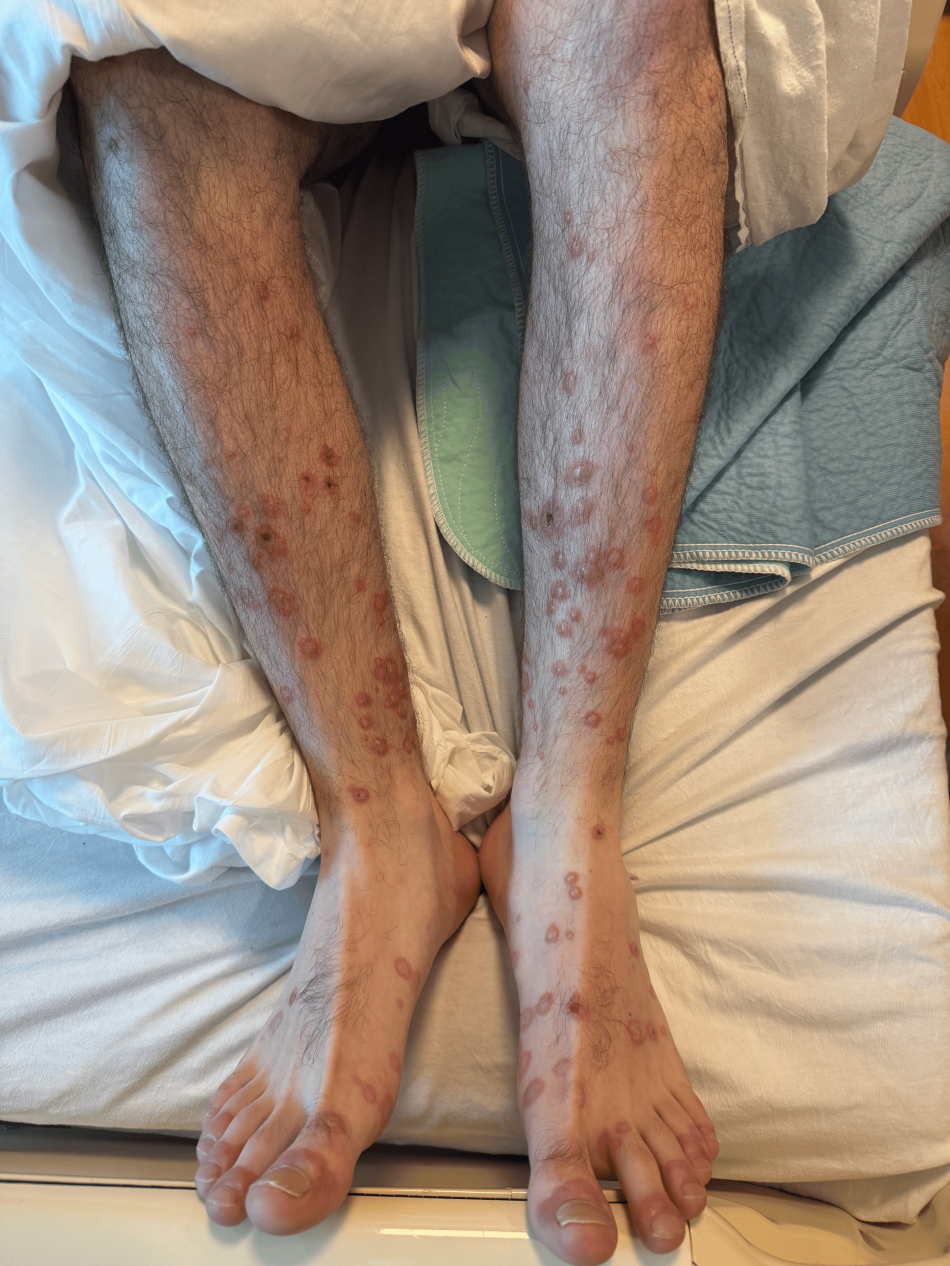
Circular vesiculobullous lesions with areas of central crusting on the bilateral lower extremities, characteristic of MIRM MIRM: *Mycoplasma pneumoniae*-induced rash and mucositis

**Figure 2:**
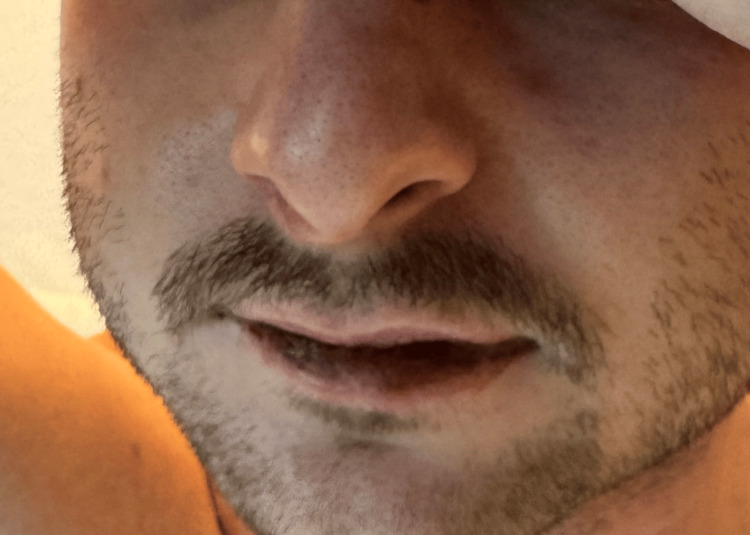
Oral mucosal involvement demonstrating erosions and mucositis commonly seen in MIRM MIRM: *Mycoplasma pneumoniae*-induced rash and mucositis

**Figure 3:**
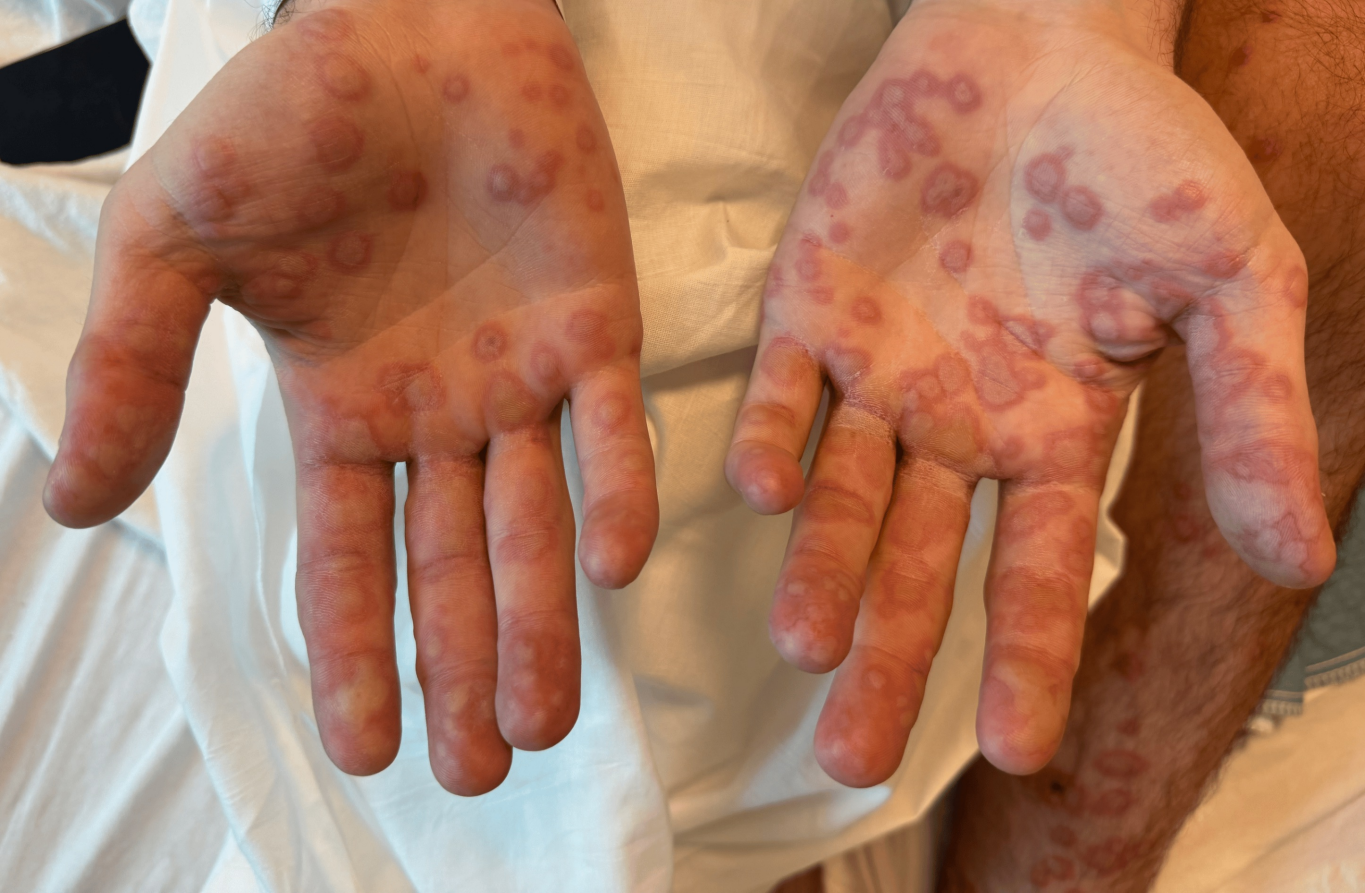
Circular vesiculobullous lesions on the bilateral upper extremities

On presentation, he was febrile to 101.5°F and tachycardic. Physical examination revealed widespread vesiculobullous rash, prominent on the hands, feet, and trunk, and oral mucositis (Figures 1-3). There was no skin sloughing, and Nikolsky's sign was negative.

Initial laboratory studies in the emergency room showed a white blood cell count of 10.2×10⁹/L with neutrophilia and relative lymphopenia. Comprehensive metabolic panel (CMP) and lactic acid were within normal limits. Urinalysis was unremarkable. Inflammatory markers C-reactive protein (CRP) and erythrocyte sedimentation rate (ESR) were elevated, consistent with systemic inflammation. At that time, blood cultures were obtained. The patient was then admitted for further evaluation. Infectious Diseases and Ophthalmology were consulted.

A respiratory pathogen PCR panel (RVP) was performed, which included testing for COVID-19, influenza, respiratory syncytial virus (RSV), and *M. pneumoniae*. Human immunodeficiency virus (HIV) antigen/antibody and *Treponema pallidum* antibody were also obtained and were negative. Due to the distribution of vesicular lesions, monkeypox PCR was obtained as well. The RVP was negative for viral pathogens but returned positive for *M. pneumoniae*.

Based on clinical presentation and laboratory results, a diagnosis of MIRM was made. Initially, the patient was started on azithromycin empirically and subsequently transitioned to doxycycline. Systemic corticosteroids were initiated to reduce mucocutaneous inflammation.

On the ophthalmologic exam, blistering epithelial changes without corneal defects were noted. Ophthalmology then recommended erythromycin ophthalmic ointment nightly, ofloxacin drops three times daily, and frequent artificial tears.

Over four hospital days, the patient's rash, oral ulcerations, and conjunctivitis demonstrated clinical improvement. This improvement was based on functional recovery, including tolerance of oral intake, downtrending lab values, serial physical examinations, and patient-reported symptom resolution. Follow-up imaging of lesion resolution was not obtained, as the patient was discharged once clinically stable. He was discharged on doxycycline to complete a 10-day course, prednisone 40 mg daily for five days, and topical ophthalmic therapies. Outpatient follow-up with Infectious Diseases and Ophthalmology were arranged.

## Discussion

*M. pneumoniae *is a relatively frequent cause of community-acquired pneumonia and is well-known for its various extrapulmonary manifestations, which can present in up to 25% of infections [6]. Among these extrapulmonary manifestations, mucocutaneous involvement is one of the most clinically important features. While previously recognized under erythema multiforme (EM) or SJS, this entity is now recognized separately as a distinct diagnosis of MIRM. Since its first definition in 2015, MIRM has been increasingly recognized in the pediatric and young adult populations [2].

MIRM is a post-infectious, likely immune-mediated condition following upper respiratory infection, particularly in adolescents and young adults [3,4]. The precise mechanism is unknown; however, T-cell immune response and immune complex deposition secondary to *M. pneumoniae *antigens have been suggested [2]. Mucocutaneous manifestations typically develop several days to one week after the onset of respiratory symptoms, which can create diagnostic uncertainty during early presentation [3].

This case highlights several important aspects of MIRM. The patient, a previously healthy male adult without significant past medical history, presented with seemingly indolent antecedent respiratory symptoms, which were subsequently followed by a rapid onset of painful mucositis, conjunctivitis, and vesiculobullous skin lesions. This clinical picture was consistent with MIRM and further supported by positive *M. pneumoniae *PCR testing. His clinical improvement with doxycycline and corticosteroids may suggest that antimicrobial therapy, in addition to supportive and anti-inflammatory measures, can lead to favorable outcomes. However, whether antibiotic treatment shortens the course of the disease is yet to be determined. In a review of 202 patients with MIRM, 35% of patients received systemic corticosteroids; although evidence currently to support this approach is weak, a short course of prednisone 1 mg/kg/day is suggested in patients with extensive mucosal involvement and severe symptoms [2].

The initial presentation of this patient raised concern for other vesiculobullous and mucocutaneous syndromes, which include SJS, TEN, and viral exanthems such as herpes simplex virus (HSV), varicella zoster virus (VZV), coxsackievirus, monkeypox, and syphilis. Distinguishing MIRM from SJS and TEN is clinically important. In SJS/TEN, extensive epidermal necrosis and skin detachment are commonly seen, whereas in MIRM, mucositis is often disproportionally more severe relative to its cutaneous manifestation [2,7]. Additionally, Nikolsky's sign is often absent in cases of MIRM, as seen in our patient. SJS/TEN is also strongly associated with recent medication exposure, which was absent in this case. Other viral etiologies were systematically ruled out with PCR and serologies, with the detection of *M. pneumoniae *as the suspected infectious trigger.

The exact immunopathology of MIRM is yet to be fully understood. A proposed mechanism includes immune complex deposition and T-cell-mediated cytotoxicity triggered by cross-reactive antigens of *M. pneumoniae *[1]. The immune response elicited is thought to cause the localized mucocutaneous inflammation without the widespread keratinocyte apoptosis that is often seen in cases of SJS/TEN [8]. Additionally, MIRM tends to follow a relatively benign course, which suggests a qualitatively different immune mechanism in comparison to hypersensitivity reactions associated with drug use.

Regarding treatment considerations, there are currently no established evidence-based standardized treatment guidelines for MIRM. In the acute settings, it may be difficult for clinicians to differentiate MIRM from other severe mucocutaneous reactions. The initial management in patients with suspected SJS/TEN involves immediate in-hospital evaluation for diagnosis confirmation, the evaluation of severity, consultation with specialists, and initiation of supportive management [7]. Supportive care, including pain management, hydration, and nutritional support, is critical especially in patients with oral and pharyngeal involvement such as ours. Corticosteroids and IVIG have been reported in severe cases, and although evidence remains limited at this time, some evidence suggests that they may hasten the resolution of systemic inflammatory symptoms and mucositis [8]. In this case, our patient improved with a short course of systemic steroids without the need for additional IVIG therapy; however, further research is needed to establish standardized guidelines for MIRM, as evidence supporting corticosteroid use remains limited.

Early recognition and ophthalmology consultation can be essential in preventing potential long-term sequelae. In this case, ocular findings were consistent with blistering epithelial disorder, and no epithelial defects or internal ocular pathology were noted on exam. Ocular symptoms resolved with topical therapy.

## Conclusions

MIRM is an increasingly recognized clinical entity. Early differentiation of MIRM from SJS and TEN is critical, as these conditions differ significantly in prognosis and management. Unlike SJS/TEN, MIRM typically follows a relatively mild clinical course with low recurrence and minimal long-term morbidity and rare mortality. This case highlights the importance of maintaining a high index of suspicion for MIRM in patients presenting with prominent mucosal involvement following a respiratory illness, especially in the absence of recent medication exposure. Management of MIRM is primarily supportive and should include targeted antimicrobial therapy for *M. pneumoniae *and early ophthalmologic evaluation if ocular involvement is suspected. Adjunctive systemic corticosteroids can be considered in severe cases with significant mucosal inflammation. This case adds to the growing body of literature describing the clinical spectrum of MIRM in young adults and highlights favorable outcomes with timely recognition and appropriate supportive care.
